# Physical, Chemical, Mechanical, and Biological Properties of Four Different Commercial Root-End Filling Materials: A Comparative Study

**DOI:** 10.3390/ma14071693

**Published:** 2021-03-30

**Authors:** Tae-Yun Kang, Ji-Won Choi, Kyoung-Jin Seo, Kwang-Mahn Kim, Jae-Sung Kwon

**Affiliations:** 1 Department and Research Institute of Dental Biomaterials and Bioengineering, Yonsei University College of Dentistry, Seoul 03722, Korea; tykang@yuhs.ac (T.-Y.K.); den0424@yuhs.ac (J.-W.C.); kjseo@yuhs.ac (K.-J.S.); kmkim@yuhs.ac (K.-M.K.); 2 BK21 FOUR Project, Yonsei University College of Dentistry, Seoul 03722, Korea

**Keywords:** mineral trioxide aggregate (MTA), root-end filling materials, endodontic materials, retrograde filling materials

## Abstract

Commercial mineral trioxide aggregate (MTA) materials such as Endocem MTA (EC), Dia-Root Bio MTA (DR), RetroMTA (RM), and ProRoot MTA (PR) are increasingly used as root-end filling materials. The aim of this study was to assess and compare the physicochemical and mechanical properties and cytotoxicity of these MTAs. The film thicknesses of EC and DR were considerably less than that of PR; however, RM’s film thickness was greater than that of PR. In addition, the setting times of EC, DR, and RM were shorter than that of PR (*p* < 0.05). The solubility was not significantly different among all groups. The three relatively new MTA groups (EC, DR, and RM) exhibited a significant difference in pH variation and calcium ion release relative to the PR group (*p* < 0.05). The radiopacity of the three new MTAs was considerably less than that of PR. The mechanical strength of RM was not significantly different from that of PR (*p* > 0.05); however, the EC and DR groups were not as strong as PR (*p* < 0.05). All MTA groups revealed cytocompatibility. In conclusion, the results of this study confirmed that EC, RM, DR, and PR exhibit clinically acceptable physicochemical and mechanical properties and cell cytotoxicity.

## 1. Introduction

Calcium silicate-based cements (CSCs) have various indications for use in endodontics, and their clinical applications have increased over the years. Mineral trioxide aggregate (MTA) is a calcium silicate-based material commonly considered ideal for endodontic treatment due to its excellent biological and physicochemical properties [1,2,3,4]. The first commercially available MTA, Pro Root MTA (Dentsply, Tulsa, OK, USA), is composed of Portland cement and bismuth oxide [5,6]. 

MTA is recommended for a number of clinical applications in endodontic treatment, such as pulp capping, pulpotomy, apexification, apicogenesis, apical barrier, repair of root perforations, formation in teeth with necrotic pulps and open apexes, root-end filling, and orthograde root canal filling [1,4]. An ideal endodontic repair material should be biocompatible, easy to handle, insoluble in body fluids, economical, and dimensionally stable for long-term clinical success [1,4]. MTA has several advantages in terms of biocompatibility, bioactivity, sealing ability, and dimensional stability [7,8].

Conventional MTA, ProRoot MTA (PR), has supplanted other endodontic materials because of its superior physico-chemical and biological properties that are due to its composition of fine hydrophilic powders of tricalcium silicate, tricalcium aluminate, tricalcium oxide, and other oxides [1,4,9]. Although it has a variety of favorable properties, conventional MTA (PR) has been reported to have several drawbacks in clinical settings because of its long setting time, expense, sensitivity to the presence of moisture, and low compressive strength compared with other endodontic treatments [1,4,10,11,12,13]. Since, PR sets relatively slowly, it may be washed out from the preparation under hemorrhagic conditions when it is used as a root-end filling material, which may cause treatment failure [1,9]. PR also has a discoloration potential, which can contribute to tooth discoloration, discoloration and it contains a variety of toxic elements. It is important to overcome conventional MTA’s biocompatibility drawbacks [1,9,14].

Recently, a new type of silicate-based MTA (Endocem MTA, Retro MTA, and Diaroot BioMTA) was developed to overcome these disadvantages when used as an endodontic treatment [7,15,16]. Endocem MTA (EC) contains fine particles of pozzolan, a calcium silicate-based material that reacts with calcium hydroxide (formed after a hydration reaction) and produces additional cementitious materials. EC has been reported to show biocompatibility similar to MTA, improve washout resistance, and cause less tooth discoloration [7,9,17]. Retro MTA (RM) was recently developed and introduced to the market as a new hydraulic calcium silicate-based material proposed for use in endodontic applications similar to those of MTA [18]. RM is a fast-setting MTA that consists of a calcium zirconia complex. According to the manufacturers, RM has less discoloration potential, is relatively cost-effective, and is easy to manipulate. Recently, a study showed that RM has similar biocompatibility to PR [15,16,19,20,21,22,23]. Dia Root Bio MTA (DR) is a newly improved calcium silicate-based MTA that is easy to manipulate and promotes cementogenesis and sealing capability inside the root canal system. However, only a limited amount of information is available on this product. Despite the increase in the use of MTA as an endodontic treatment material, there has been limited information on these new products in the previous literature [16].

A review of previous studies revealed no direct comparisons of these three commercial MTAs with PR. Thus, the purpose of this study was to assess and compare the physicochemical, mechanical properties, and cytotoxicity of these three new types of MTA with Pro Root MTA as a retrograde filling material. In the present study, we compared the three commercial MTAs with Pro Root MTA in terms of setting time, film thickness, solubility, radiopacity, pH, the release of calcium ions, compressive strength, and cytotoxicity.

The null hypotheses of this study were; (1) There will be no significant differences in the physicochemical properties between any of the three new MTAs in comparison to PR, (2) There will be no significant differences in the mechanical properties between any of the three new MTAs in comparison to PR, (3) There will be no significant differences in the cytotoxicity between any of the three new MTAs in comparison to PR.

## 2. Materials and Methods

### 2.1. Materials and Preparation of the Specimens

Four commercially available MTAs were used in the present study (Table 1). Endo-cem MTA (EC) was mixed with sterile normal saline at a W/P ratio of 0.12 cc:300 mg, Dia-Root Bio MTA (DR) was mixed with sterile distilled water at a W/P ratio of 0.225 g:0.5 g, Retro MTA (RM) was mixed with provided liquid at a W/P ratio of 3 drops:0.3 g, and Pro Root MTA (PR) was mixed with provided liquid at a W/P ratio of 3:1 as recommended instructions by the manufacturer. In addition, sterile normal saline (Cleancle, JW Pharmaceutical Co., Seoul, Korea) and sterile distilled water (sterilized distilled water for intermediate and external perfusion, JW Pharmaceutical Co., Seoul, Korea) were used to mix EC and DR, respectively.

### 2.2. Film Thickness, Setting Time, and Solubility

The film thickness, setting time and solubility tests of the materials were measured based on the International Organization for Standardization (ISO) 6876 standard methods. To investigate the film thickness, two flat glass plates with 25 mm square (contact surface area of approximately 625 ± 50 mm^2^) were combined to measure the thickness of the two glass plates in contact. Three minutes after starting the mixing, a load of 150 N was applied vertically on the upper plate. Ten minutes after the start of mixing, the thickness to an accuracy of 1 μm of the space between the two glass plates that was filled with experimental material was measured using a digital caliper (Mitutoyo Model CD-15CPX; Mitutoyo Co., Kawasaki, Japan). The test was repeated 3 times for each group.

To analyze the setting time, the initial and final setting times were measured by evaluating the absence of indentations caused by Gillmore needles. The mixed experimental material was filled into a mold with internal diameter of 20 mm and height of 2 mm. Before the test, all the apparatus was conditioned for 24 h under 100% relative humidity at 37 ± 1 °C. The setting time test was performed under 100% relative humidity at a temperature of 37 ± 1 °C using the Gillmore needle. Each of the two indenters with flat ends and a mass of 100 ± 0.5 g (initial setting time) or 453.6 g (final setting time) were loaded vertically onto the top surface of the specimens. The test was repeated 3 times for each group.

To measure the solubility, the mixed experimental material was placed in a mold with 20 mm diameter and 2 mm height, and the excess was removed. The filled mold was placed under 100% relative humidity at 37 ± 1 °C for 7 d. For each tested experimental material, two specimens were used (total 6, n = 3). The initial mass of the two specimens (m_0_) and the container (M_0_) were measured to the nearest 0.001 g by an analytical balance (XS105, Mettlertoledo AG, Greifensee, Switzerland). The two specimens were immersed in 50 mL distilled water and placed in a water bath maintained at 37 ± 1 °C for 24 h. After 24 h, the specimen was removed from the container, washed with distilled water, and then placed in an oven at 80 ± 2 °C for drying. Subsequently, the desiccator was cooled and weighed to determine the final mass (M_1_). The final mass of each specimen was deter-mined, and the loss of mass was calculated by the following equation:

$$\mathrm{Solubility}\;\left(\%\right)=100\times \left(M_{1}-M_{0}\right)/m_{0}$$

### 2.3. Radiopacity

Radiopacity evaluation of the set materials was performed using the ISO 6876 and 13116. Each mixed MTA material was filled into a mold 10.0 ± 1.0 mm in diameter and 1.0 ± 0.1 mm height. To obtain the radiographic images, both specimens and an aluminum step wedge were placed on a digital sensor and exposed to an X-ray unit (Carestream CS7600, Siemens, Munich, Germany) at 65 ± 5 kV and 10 mA with a 300 ± 100 mm focus-film distance. The grey pixel values of each specimen were determined using the Photoshop program (Adobe, San Jose, CA, USA), and the equivalent radiopacity of the cement sample was calculated in mm of aluminum (Al mm).

### 2.4. Compressive Strength at 7 Days

Each MTA materials was mixed and filled into a mold 4 mm in diameter and 8 mm in height. Seven cylindrical samples for each group were prepared. The specimens were incubated under 100% relative humidity at a temperature of 37 ± 1 °C. The specimens for compressive strength were ground with a wet 600 grit 7 d after preparation. A computer-controlled universal testing machine (Model 3366; Instron^®^, Norwood, MA, USA) was used to compress the specimens. The compressive strength was measured at a crosshead speed of 0.25 mm/min.

The maximum load of the compressive strength was recorded and calculated in MPa as:

$$\mathrm{CS}=4P/\pi D^{2}$$

where CS is the compressive strength, *p* is the maximum force applied in Newtons (N), and *D* is the mean diameter of the specimen in millimeters (mm).

### 2.5. pH, Calcium Ion Release, and Bioactivity

To analyze the pH variation of the soaking solution and calcium ion release, each MTA material was prepared and filled into a mold with an internal diameter of 10 mm and a height of 1 mm. After filling, the specimen was stored at 37 ± 1 °C for 24 h. The specimen was separated from the mold and was then immersed in 10 mL of Hank’s balanced salt solution (HBSS; H6648, Sigma Aldrich, St. Louis, MO, USA) [24]. A pH meter (Orion 4 Star, Thermo Fisher Scientific Inc., Singapore) calibrated using buffer solutions of pH 4.01, 7.00, and 10.01 was used. The pH variation of the HBSS-immersed specimen was measured at 3 h, 6 h, 24 h, 72 h, and 168 h.

The same solutions used to test the pH variation were used to test for calcium ion release. After 168 h, to analyze calcium ion release from the specimens, the HBSS was filtered with a 0.22-μm syringe filter (DISMIC 25CS, Advantec, Osaka, Japan) and subjected to inductively coupled plasma optical emission spectrometry (ICP-OES, Optima 8300, PerkinElmer, Waltham, MA, USA). The measurements of the pH and calcium ion release were repeated 3 times, and mean and standard deviations were used.

The morphology of the specimens before and after immersion in HBSS solution was analyzed by field emission scanning electron microscopy (FE-SEM, MERLIN, Carl Zeiss, Oberkochen, Germany) after ion sputtering (Leica EM ACE600) to coat the specimen with platinum. 

### 2.6. Cell Viability

Mouse fibroblast cells (L929; CRL-6364; ATCC, Manassas, VA, USA) were cultured in RPMI 1640 (Welgene, Daegu, Korea) supplemented with 10% fetal bovine serum and 1% antibiotics under standard cell culture conditions (37 °C, 100% humidity, 95% air, 5% CO_2_). L929 cells were seeded in a 96-well plate at a density of 1 × 10^4^ cells/well and incubated for 24 h under standard cell culture conditions. 

The specimen of each MTA material was formed under aseptic conditions in a sterile cylindrical mold 5 mm in diameter and 2 mm high and sterilized using ultraviolet irradiation (UV) for 30 min before storage in an incubator at 37 ± 1 °C for 24 h to achieve complete setting. The ratio of material surface area to medium volume was set at approximately 3 cm^2^/mL in accordance with the ISO 10993-5 and 12 [25,26]. 

The extraction medium was filtered through a 0.22 μm syringe filter, and three concentrations (50%, 25%, and 12.5%) were prepared and applied to the cells. At 24 h, the cytotoxicity was determined using a 3-(4,5-dimethylthiazol-2-yl)-2,5-diphenyltetrazolium bromide (MTT; Sigma-Aldrich, St. Louis, MO, USA) assay. MTT solution was added to the cells and incubated at 37 °C for 2 h in the dark. The MTT solution was then removed, and 100 μL of dimethyl sulfoxide (0231, VWR Life Science, Radnor, PA, USA) was added to each well. The optical density (OD) at 570 nm was measured using a plate reader (Epoch, BioTek, Winooski, VT, USA). The experiments were performed in triplicate.

### 2.7. Statistical Analysis

The results of film thickness, setting time, solubility, radiopacity, compressive strength, pH variation, calcium ion release, and cell viability were analyzed using the SPSS 25 software program (IBM Corp., Armonk, NY, USA). To calculate the mean and standard deviation (SD), descriptive statistics was conducted. In addition, to analyze the significant difference among the MTA groups, one-way analysis of variance (ANOVA) and Tukey’s honest significant difference (HSD) test were performed. *p*-vales less than 0.05 were considered statistically significant.

## 3. Results

### 3.1. Film Thickness, Setting Time, and Solubility

Table 2 shows the means, standard deviations, and statistical comparisons of the film thickness, setting time, and solubility tests of the commercial materials studied.

The mean film thickness values of EC, DR, RM, and PR were 0.28, 0.26, 0.96, and 0.58 mm, respectively. RM had the thickest film, and EC and DR had the thinnest films among the tested groups (*p* < 0.05).

Regarding initial setting times, RM had the shortest initial setting time, and DR had the longest setting time (*p* < 0.05). Additionally, RM had the shortest final setting time, and PR had the longest final setting time (*p* < 0.05). RM presented the lowest mean final setting time values among the materials tested (*p* < 0.05), followed by EC, DR, and PR (*p* < 0.05).

For the results of the solubility test, no significant differences were observed among the evaluated materials (*p* > 0.05). EC showed the highest value over 3% (8.11%).

### 3.2. pH Variation, Calcium Ion Release, and Bioactivity

The means, standard deviations, and statistical comparisons for pH and calcium ion release (mg/L) are shown in Table 3. The pH values measured for DR were slightly higher at all time points. PR had a lower pH value than the other groups during the initial period (3, 6, and 24 h). After 72 h, all of the materials had similar pH values, except for EC, which showed lower pH values (*p* < 0.05). DR and RM had the highest pH, followed by PR and EC, at 168 h (*p* < 0.05). Only RM showed a statistically significant difference in relation to the interaction between the storage solution and materials at all times (*p* < 0.05). There was no significant difference between the pH values obtained for all groups at 168 h immersion time (*p* > 0.05).

With regard to the release of calcium ions, all materials released considerable amounts at 7 days. Additionally, the results of the ICP-OES analysis showed that RM and PR had significantly more calcium release than EC and DR (*p* < 0.05). 

**Figure 1:**
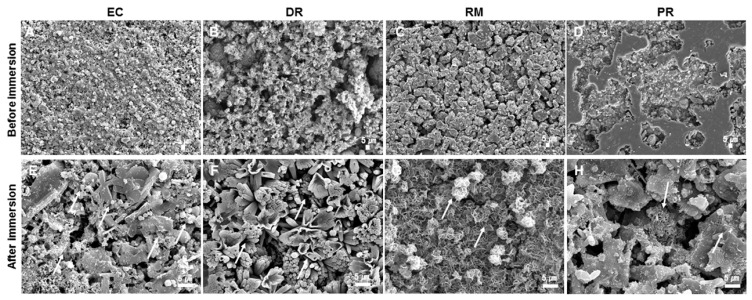
Scanning electron microscopy analysis of (**A**) Endocem MTA (EC), (**B**) Dia-Root Bio MTA (DR), (**C**) Retro MTA (RM), and (**D**) Pro Root MTA (PR) before immersion in Hank’s balanced salt solution (HBSS) (500× magnification). Surface precipitates on sets (**E**) EC, (**F**) DR, (**G**) RM, and (**H**) PR produced after immersion in HBSS for 7 d (2000× magnification). Scale bar represents 5 μm.

The morphology of the surfaces formed after the pH test of each of the samples can be assessed using the SEM images presented in Figure 1. SEM analysis revealed the presence of precipitates with various morphologies (Figure 1). All specimens had prismatic, hexagonal, cubical, needle-like, globular-like, petal-like, and scale-like crystalline precipitates on their surface (Figure 1A–D), which were not revealed in specimens that had not been immersed in HBSS (Figure 1E–H).

### 3.3. Radiopacity

**Figure 2:**
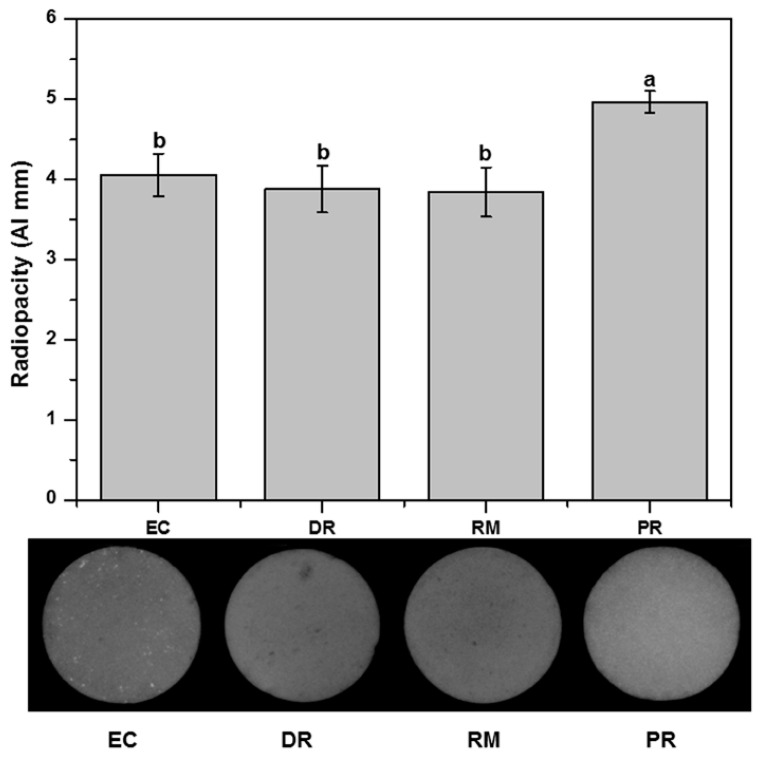
The radiopacity of the specimens was expressed as the equivalent thickness of aluminum (mm Al), and radiographic images of the specimens were obtained. The same lowercase letters indicate statistically significant differences (*p* < 0.05).

The results for the radiopacity values are presented in Figure 2. All MTA groups achieved the minimum required radiopacity value of 3 mm of Al, as recommended by the ISO 6876 standard. PR showed the highest radiopacity values among the tested materials (*p* < 0.05), equivalent to 4.97 Al mm. The EC, DR, and RM groups were not significantly different among the tested MTA groups regarding their radiopacity (*p* > 0.05). The radiopacity of EC, DR, and RM were equivalent to 4.06, 3.88, and 3.84 Al mm, respectively.

### 3.4. Compressive Strength at 7 Days

After 7 d of setting, the specimens were tested for compressive strength (MPa). The results for the mechanical properties of the specimens are presented in Table 2. Overall, the compressive strength values of PR (mean = 62.70 ± 15.92 MPa) and RM (mean = 77.48 ± 22.49 MPa) were significantly greater than those of EC (mean = 18.51 ± 9.18 MPa, *p* < 0.05) and DR (mean = 23.45 ± 9.65 MPa, *p* < 0.05). PR was not significantly different in compressive strength from RM (*p* > 0.05), and EC was not significantly different in compressive strength from DR (*p* > 0.05).

### 3.5. Cell Cytotoxicity

**Figure 3:**
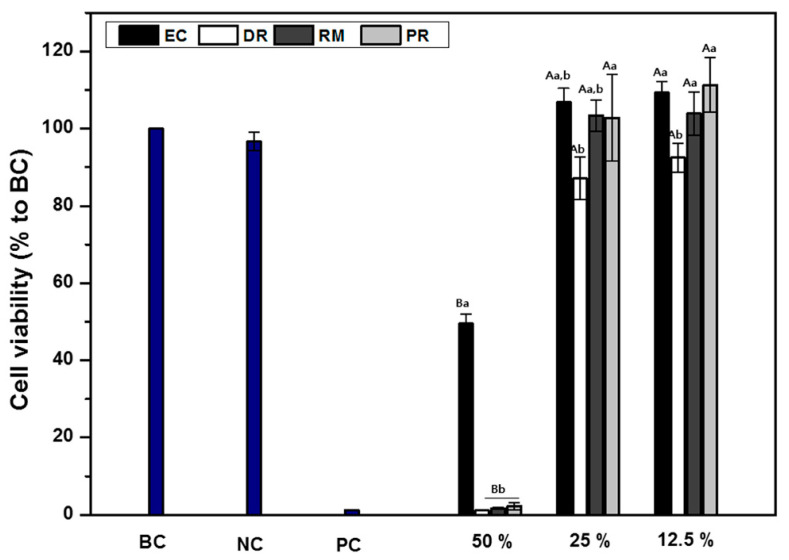
Cytotoxicity with the extracts from commercial MTA as measured by the MTT assay. The same uppercase letters indicate statistically significant differences in the dilution factor within each material (*p* < 0.05). The same lowercase letters indicate statistically significant differences within each MTA group (*p* < 0.05). BC, blank control; NC, negative control; PC, positive control.

The cell cytotoxicity of the extract from four MTA specimens at different extract concentrations is shown in Figure 3. The cells cultured on MTA eluates for 24 h were measured by MTT assays, considering cells cultured in the absence of the specimen extract as a blank control. When all specimen extract concentrations were diluted to 50%, an effect on fibroblast cell cytotoxicity was detected (below 70%), and when the extract concentration was diluted to 25% or lower, no effect on cell viability was shown (above 70%). EC eluents, diluted 50%, exhibited higher cell viability than the other MTA groups (*p* < 0.05). The viability of cells treated with EC, RM, and PR was similar in the 25 and 12.5% diluted extracts (*p* > 0.05). The viability of cells treated with DR was significantly lower than those treated with PR in 25 and 12.5% diluted extracts (*p* < 0.05), whereas there was no significant difference in cell viability compared with EC and RM (*p* > 0.05). Additionally, for DR, cells incubated in 12.5% diluted extract had significantly lower cell viability than cells incubated at the same extract concentrations of the other groups (*p* < 0.05). Cell viability was significantly affected in the presence of a dilution factor between 50% and 25% (*p* < 0.05); however, there was no significant difference between 25% and 12.5% (*p* < 0.05). For all MTA groups, significant differences were detected between the 50% and 25% dilutions (*p* < 0.05). As expected, extract dilution in medium decreased MTA cytotoxicity.

## 4. Discussion

When nonsurgical endodontic treatment fails or cannot be performed, surgical root canal treatment should be conducted. This surgical procedure includes the placement of a retrograde filling material in close contact with the peri-radicular tissue. Therefore, the physical and chemical properties as well as the biocompatibility of the retrograde filling material are very important for the success of apical surgery [26]. Generally, MTA is considered the gold standard material in clinical applications. Several previous studies found that MTA as a root-end filling material had excellent physicochemical properties and demonstrated its supremacy over other commonly used materials [26,27,28].

Recently, several new MTA-based endodontic treatment materials were introduced to improve clinical practice [9,13,17,29,30,31]. We evaluated the physicochemical and mechanical properties and cell cytotoxicity of three new commercial MTAs in comparison with conventional MTA (PR).

The physical properties of MTA, such as setting time, film thickness, and solubility, strongly affect the material’s clinical performance. In particular, these properties are important clinical factors affecting their sealing ability. For instance, the thicker the film, the lower the chance of material penetration into the accessory root canal system [32]. Several previous works reported that a thinner layer of sealer positively affected the sealing ability of the root canal filling [33,34,35]. In this study, the results showed that all MTA groups had a thinner film than PR (*p* < 0.05), except for RM (*p* > 0.05).

A long setting time of materials provides an adequate working time when performing surgical treatment, such as retrograde filling or perforation repairs. However, in certain clinical situations, such as apexification and particularly apical surgery, unset material may be washed out by body fluid and/or blood in the surgical field, which may lead to treatment failure and cytotoxicity [36,37,38,39].

A fast setting time for materials ensures that the MTA has the least amount of interaction time with the contaminants present in the oral cavity, making it easier to place a second restorative material on top of the MTA [36,40,41]. The MTA setting time could be a factor that is directly related to surgical root canal treatment success [39]. Hence, in some clinical conditions, an accelerated setting time is required to avoid dissolution of the materials under oral conditions [36]. The results of this study confirmed that the final setting time of the three new MTAs was shorter than that of PR (*p* < 0.05). A previous study reported that the proper setting time is considered to be 10 and 15 min in clinical situations [36]. This result is in agreement with a previous setting time test and indicates that both EC and RM had the proper setting time [7,15,16,18]. The main advantage of RM over PR includes its reduced setting time because of the fast setting of its calcium silicate-based materials, which form calcium zirconia complexes [15,16]. The EC group sets quickly without the addition of a chemical accelerator because it contains fine-particle pozzolan cement [6,42].

Solubility is an acceptable property for endodontic treatment materials, since it allows the release of ions. However, it is important that excess solubilization of the material does not occur [43]. Most endodontic failures occur as a result of the leakage of irritants from pathologically involved root canals into the periapical tissues [44]. Hence, endodontic and restorative materials should also have a long-term seal and prevent leakage from the oral cavity and/or the periapical tissue [45]. To provide long-term stability and prevent microleakage from the periapical tissue, root-end filling material must have a low solubility [44]. Thus, a low solubility in distilled water, as proposed in the Standard of the International Standard Organization (ISO) 6876, is required [46]. Following this test, the weight loss of each specimen is indicated as the percentage of the original mass, and the ideal recommendation is a value less than 3% [45,46].

Radiopacity is a very important characteristic required for pulp treatment materials [47]. Root-end filling and endodontic repair materials must have radiopacity to allow for evaluation of the quality of the filling for patient safety. A radiopaque material is essential to identify the location of the material in the root canal and to allow for filling failures to be corrected before final restoration [48]. MTA with a radiopacity value lower than 3 mm Al is hardly distinguishable from dentine. Clinically acceptable values of radiopacity, i.e., higher than 3 mm Al, are mandatory for controlling the quality of root canal filling [43,49]. In the present study, all MTA groups achieved the minimum required radiopacity value of 3 mm of Al, as recommended by the ISO 6876 standard.

When MTA contacts fluids, it rapidly releases calcium and hydroxyl ions and creates an alkaline pH on its external surface, leading to the nucleation and crystallization of apatite on the material’s surface [50,51]. Numerous previous studies reported that MTA has the ability to form calcium phosphate apatite crystals on its surface after contact with phosphate-containing simulated body fluid solution [50,51,52]. Consequently, the deposition of calcium and phosphate apatite into voids and spaces between the dentin, root canal systems, and root filling material enables MTA to encourage regeneration and remineralization of adjacent hard tissues while also improving its sealing capacity [50,53,54]. Thus, apatite-forming ability may provide clinical advantages by improving the sealing via the deposition of apatite at the interface and inside the dentinal tubules of the root canal when MTA is used as a root canal filling material [13,50,54]. HBSS solution was used in the present study as a storage solution to simulate the clinical environment. In this study, all materials showed alkalinizing activity and the formation of crystalline apatite on the surface of the specimens.

MTA is a hydraulic cement consisting of fine hydrophilic particles that gradually harden in a wet environment [11,55]. The compressive strength of MTA which was set for 28 days is considered an indicator of the progression of the hydration reaction and a reflection of the setting process. In this study, compressive strength of each sample was measured following 7 days of storage. The number of days of storage was determined in accordance to a draft of ISO 6876 that is currently under revision and in order to compare MTA following adequate hydration. Still, the study is limited as the optimal days of storage were not predetermined which would have resulted in a higher level of compressive strength. In accordance with clinical perspectives, a greater compressive strength of MTA is considered to be an important feature when this material is used as pulp capping or as a coronal restorative material when it is submitted to occlusal and mastication forces [56]. However, when MTA is used as a root-end filling material, where minimal forces are applied, a low compressive strength will not be a major clinical drawback [40,57].

Cell cytotoxicity of endodontic materials is of great concern because irritation of the surrounding tissue can affect periapical tissue regeneration [22,58,59]. In an in vitro study, after application in medium, MTA suffers a hydration reaction that results in the formation of calcium hydroxide and subsequent ionic dissociation into calcium and hydroxyl ions, which is responsible for an increase in pH value and an elevated calcium concentration in the cell medium [60]. In this study, the four commercial MTAs maintained a cell viability rate above 70% for all dilutions, except for the 50% diluted eluent. When comparing the cell cytotoxicity of the four commercial MTAs in this study, PR resulted in the highest cell viability, and DR was significantly more cytotoxic than the other two types (*p* < 0.05). This cytotoxic effect of DR may be attributed to the differences in the initial amount of various ions released from the materials. Additionally, another factor may be caused by the characteristics of the DR material itself, which can increase the pH value. According to the manufacturer, DR has a strong antibacterial effect with a high alkaline pH (above pH 12). However, the DR group achieved the minimum required recommendation of a 70% relative cell viability rate, as recommended by the ISO 10993-5 standard.

In terms of the initial null hypotheses of this study, the hypothesis about their physicochemical properties was partially accepted. When the PR group was compared with the EC, DR, and RM groups, the results of this study showed statistically significant differences in film thickness, setting time, pH variation, calcium ion release, and radiopacity (*p* < 0.05), although no significant differences were found for the solubility (*p* > 0.05). However, their solubility was not significantly different from that of the PR group (*p* > 0.05). The second null hypothesis was partially rejected because the EC and DR groups revealed a significant difference in compressive strength (*p* < 0.05) when compared to the PR group; however, the RM group revealed no significant difference compared to PR (*p* > 0.05). Finally, when the three new MTAs were compared with PR, the results of this study showed a statistically significant difference in cell cytotoxicity for all dilutions (*p* < 0.05). Therefore, the third null hypothesis was rejected.

This study has several limitations. This in vitro study could not sufficiently simulate the clinical situation, which involves a complex and variable biomechanical environment. Also, as stated earlier, some of the methods are based on ISO 6876 where clinical relevance may be different between conventional root canal filling material and MTA. Thus, additional studies are needed for long-term and simulated clinical situation evaluations while such limitations are also something that would be useful when considering revision of the current version of ISO 6876.

## 5. Conclusions

In conclusion, the present study confirmed that EC, RM, and DR exhibit clinically acceptable physicochemical and mechanical properties and cell cytotoxicity relative to PR. Therefore, we confirmed that the EC, RM, and DR have the physicochemical and biocompatible characteristics that could be alternatives to conventional MTA as a retrograde filling material.

## Figures and Tables

**Table 1:** Information on the four commercially available mineral trioxide aggregates (MTAs) used in the present study.

Materials	Composition	Manufacturer
Endocem MTA (EC)	Tricalcium silicate, tricalcium aluminate, dicalcium silicate, bismuth oxide	Maruchi, Wonju, Korea
Dia-Root Bio MTA (DR)	Calcium silicate, amorphous fumed silica, zirconium dioxide	Diadent, Cheongju, Korea
Retro MTA (RM)	Calcium carbonate, silicon dioxide, aluminium oxide, calcium zirconia complex	BioMTA, Seoul, Korea
Pro Root MTA (PR)	Portland cement, bismuth oxide, calcium sulfate dihydrate, tetracalcium aluminoferrite, gypsum, calcium oxide	Dentsply, Tulsa, TN, USA

**Table 2:** Means ± standard deviation (SD) for film thickness, setting time, solubility, and compressive strength.

Materials	Film Thickness	Setting Time		Solubility	Compressive Strength
	(mm)	Initial Setting (min)	Final Setting (min)	(%)	(MPa)
EC	0.28 ± 0.16 ^a^	4.45 ± 0.05 ^b^	16.60 ± 1.15 ^b^	8.11 ± 11.40 ^a^	20.33 ± 12.54 ^b^
DR	0.26 ± 0.07 ^a^	27.24 ± 0.88 ^d^	49.31 ± 2.60 ^c^	2.23 ± 0.33 ^a^	20.64 ± 9.25 ^b^
RM	0.96 ± 0.18 ^c^	3.05 ± 0.05 ^a^	4.43 ± 0.12 ^a^	0.89 ± 0.31 ^a^	65.69 ± 27.26 ^a^
PR	0.58 ± 0.05 ^b^	18.53 ± 0.42 ^c^	122.67 ± 6.43 ^d^	0.82 ± 0.34 ^a^	68.21 ± 16.22 ^a^

Different lowercase superscript letters indicate statistically significant differences between MTA groups (*p* < 0.05).

**Table 3:** pH variation and calcium ion release (mg/L) in soaking solution for 7 days.

Materials	pH and Calcium Release in Soaking Water (Means ± SD)					
	3 h	6 h	24 h	72 h	168 h	Ca Ions Concentration at 7 Days (mg/L)
EC	11.58 ± 0.01 ^ABab^	11.63 ± 0.07 ^ABab^	11.28 ± 0.05 ^Cb^	11.49 ± 0.08 ^Bb^	11.70 ± 0.01 ^Ac^	76.33 ± 2.93 ^c^
DR	12.13 ± 0.10 ^BCa^	12.30 ± 0.06 ^ABa^	12.07 ± 0.07 ^Ca^	12.19 ± 0.05 ^ABCa^	12.33 ± 0.07 ^Aa^	377.34 ± 52.19 ^a^
RM	11.09 ± 0.09 ^Eab^	11.28 ± 0.04 ^Dbc^	11.85 ± 0.09 ^Ca^	12.13 ± 0.01 ^Ba^	12.38 ± 0.05 ^Aa^	346.78 ± 92.14 ^ab^
PR	10.20 ± 0.48 ^Cb^	10.66 ± 0.65 ^BCc^	11.42 ± 0.17 ^ABb^	12.01 ± 0.30 ^ACa^	11.97 ± 0.06 ^Ab^	215.16 ± 62.25 ^cb^

Different uppercase superscript letters indicate statistically significant differences between immersion times (*p* < 0.05, in the same line). Different lowercase superscript letters indicate statistically significant differences between MTA groups (*p* < 0.05, in the same column).

## Data Availability

Data is contained within the article.
